# Enhancing strawberry resilience to saline, alkaline, and combined stresses with light spectra: impacts on growth, enzymatic activity, nutrient uptake, and osmotic regulation

**DOI:** 10.1186/s12870-024-05755-5

**Published:** 2024-11-01

**Authors:** Mohammad Reza Malekzadeh, Hamid Reza Roosta, Hazem M. Kalaji

**Affiliations:** 1 Department of Horticultural Sciences, Faculty of Agriculture, Vali-e-Asr University of Rafsanjan, Kerman, 7718817111 Iran; 2 Department of Horticultural Sciences, Faculty of Agriculture and Natural Resources, Arak University, Arak, 38156-8-8349 Iran; 3 Department of Plant Physiology, Institute of Biology, Warsaw University of Life Sciences-SGGW, 159 Nowoursynowska Street, Warsaw, 02-776 Poland

**Keywords:** Antioxidant activity, LED, Strawberry, Stress condition

## Abstract

**Background:**

This study examines the effects of various complementary light spectra on the growth, development, antioxidant activity, and nutrient absorption in strawberry plants under stress conditions. Light-emitting diodes (LEDs) were used to provide specific wavelengths, including monochromatic blue (460 nm), monochromatic red (660 nm), a dichromatic mix of blue and red (1:3 ratio), full-spectrum white light (400–700 nm), and ambient light as a control (no LED treatment). The stress treatments applied were: control (no stress), salinity (80 mM NaCl), alkalinity (40 mM NaHCO₃), and a combined salinity/alkalinity condition.

**Results:**

Our results indicated that complementary light spectra, especially red and blue/red, helped mitigate the adverse effects of stress on plant growth and development. These spectra improved plant tolerance by enhancing the activity of polyphenol oxidase and peroxidase enzymes and increasing starch accumulation in the leaves. Furthermore, under stress conditions, red and blue-red light significantly boosted fruit anthocyanin levels. Although stress elevated antioxidant activity, supplementary light reduced this activity by alleviating stress compared to ambient light. While stress led to increased Na and Cl ion concentrations in leaves, treatments with blue, red, and blue-red light minimized these harmful effects and promoted the absorption of beneficial ions such as K, Mg, Fe, and Cu.

**Conclusions:**

Adjusting light quality significantly influences the morphology and physiology of strawberry plants, underscoring the role of specific light spectra in promoting optimal growth under stress conditions.

**Clinical trial number:**

Not applicable

## Background

Sunlight is the primary driver of plant growth and development. However, with the rise of controlled-environment agriculture (CEA) and the demand for year-round production, research on artificial lighting has gained increasing importance. McCree’s pioneering work in 1972 introduced the “action spectrum” for crop plants, illustrating how various light wavelengths influence photosynthesis [1], thereby establishing a foundation for understanding how specific light spectra can be manipulated to impact plant growth. Subsequent research highlighted the role of blue and red light in chloroplast acclimation, revealing the complex interplay between light quality and plant physiology [2]. These foundational studies paved the way for more in-depth investigations into the effects of artificial lighting on both vegetative and reproductive growth. Today, research focuses on optimizing red and blue light ratios to achieve desired outcomes. For example, a higher red/far-red light ratio can stimulate flowering, while a greater proportion of blue light promotes bushier, more compact growth [3]. This expanding body of knowledge empowers growers to tailor light spectra to their specific needs, potentially enhancing yields and improving plant quality.

LED lamps, known for their precise control over specific wavelengths, typically emit a combination of blue, red, far-red, or white light. Studies indicate that these light spectra increase photosynthetic pigments and photosynthesis rates in cherry tomatoes [4], expand leaf area [5], and enhance strawberry fruit size [6]. While greenhouses have traditionally depended on sunlight, limitations such as short winter days, abiotic stresses, and unpredictable weather conditions can hinder crop production. In response, artificial lighting has been explored for decades. Bönning’s groundbreaking work in 1956 introduced photoperiod manipulation, demonstrating that artificial lights could extend daylight hours and influence flowering times [7]. This discovery fueled further research into light quality and intensity, leading to optimized lighting environments for specific crops. Today, greenhouses utilize various artificial lighting technologies to enable year-round production and enhance yields, making greenhouse agriculture a more reliable and controlled food system [8]. The application of LED light spectra with a higher percentage of red light has been shown to increase the biomass and overall performance of strawberry plants [9]. Additionally, blue, red, and blue/red supplementary light treatments have been found to boost levels of chlorophyll, acidity, and phenolic compounds in fruits, while also promoting higher fruit yield compared to plants without supplementary lighting [10]. These findings highlight the effects of modified light conditions on strawberry fruit production and quality, emphasizing the advantages of complementary light over monochromatic light spectra in growth chambers.

Despite the controlled conditions in greenhouses, plants still encounter challenges from abiotic stresses such as extreme temperatures, nutrient imbalances, and light fluctuations. Among these, salinity and alkalinity in water, soil, or fertilizers are particularly concerning due to water reuse and reliance on specific water sources. Recent studies have underscored the negative impact of salinity on various physiological processes, including water uptake, photosynthesis, and nutrient transport, ultimately leading to reduced yields [11]. Furthermore, research has examined the interaction between salinity and alkalinity, noting that high sodium levels associated with alkalinity can exacerbate salinity’s adverse effects by impairing root function and nutrient absorption [12]. These findings emphasize the need for effective monitoring and management strategies to mitigate salinity and alkalinity stress within greenhouse settings.

While artificial lighting in greenhouses has traditionally focused on maximizing growth under limited natural light, recent research explores its potential to alleviate abiotic stresses such as salinity and alkalinity. For instance, one study demonstrated that blue and red LED light could reduce the impact of alkalinity stress on spinach by promoting root growth, enhancing nutrient uptake, and improving plant tolerance to alkaline conditions [13]. Additionally, supplementation with blue and red light has been shown to increase antioxidant compounds, further supporting optimal growth and stress resilience [14]. Plants have intricate mechanisms for coping with stress, and blue and red light play crucial roles in enhancing these mechanisms [15–17]. These findings suggest that optimizing supplemental light spectra may offer a promising strategy to bolster plant resilience against stressors like salinity and alkalinity in greenhouses, ultimately leading to higher yields and more sustainable production.

Given the growing threat of increased soil and water salinity and alkalinity to strawberry production, this study aims to explore the potential of complementary light spectra to mitigate these stressors. We investigated how different light wavelengths impact vegetative growth, reproductive parameters, enzyme activity, osmotic regulation, and nutrient uptake in strawberry plants subjected to salt and alkali stress. By assessing these factors, we seek to identify optimal light spectra that enhance plant resilience and improve strawberry performance under challenging conditions. We hypothesize that specific light spectra can enhance strawberry plant tolerance to abiotic stresses like salinity and alkalinity by modulating enzyme activity, osmotic regulation, biochemical composition, and nutrient absorption. Recognizing that these stresses often occur together in natural conditions, we also examined their combined effects alongside individual impacts.

## Materials and methods

### Plant material and growth conditions

This experiment was conducted in 2024 in the research greenhouse at Vali-e-Asr University, Rafsanjan, Iran, located at latitude 30° 21’ 17.6004’’ N and longitude 56° 0’ 9.738’’ E, at an elevation of 1545.924 m. Strawberry plants (Fragaria × ananassa Duch, cv. Sabrina) were sourced as bare-root specimens from a Namam nursery in Kurdistan, Iran. The plants were grown in 4 L pots containing a 70:30 cocopeat-to-perlite mixture, with two plants per pot. Each experimental unit comprised three pots, with a total of 60 pots used for the experiment. Greenhouse conditions were maintained at a consistent temperature of 25/15°C ± 2 °C (day/night), with a photoperiod of 11/13 hours (light/dark), and relative humidity at 50 ± 10%. Throughout the growing period, fertigation was applied using Morgan’s nutrient solution [18] with an electrical conductivity (EC) of 1.4 dS/m and a pH of 6.5.

### LED tubes and the light treatments

**Fig. 1:**
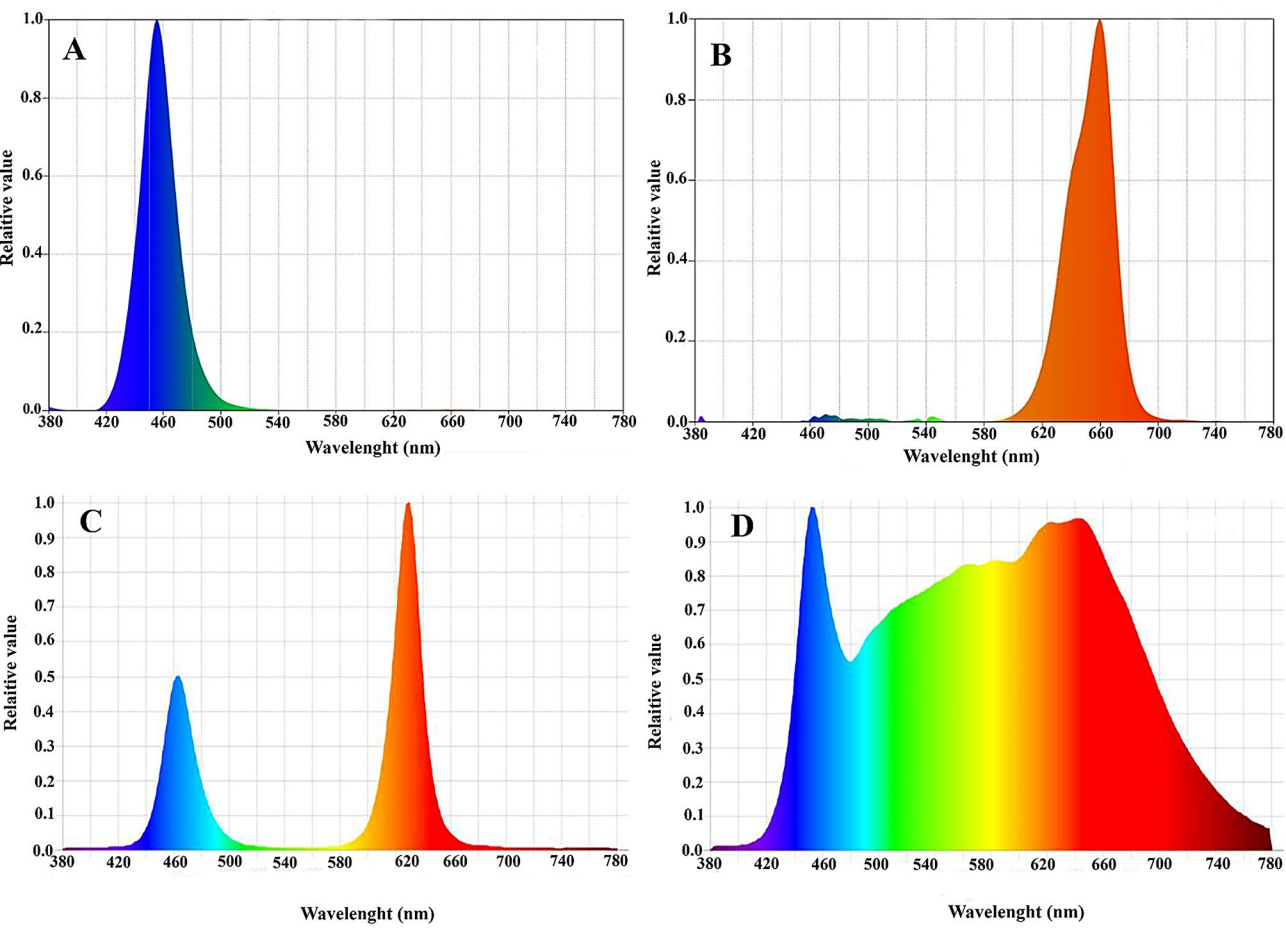
Spectral composition of different LEDs (**A**: monochromatic blue; **B**: monochromatic red; **C**: dichromatic blue/red (1:3); **D**: dichromatic white) used during the experiment

The plants were grown under light-emitting diodes (LEDs) with varying spectral ranges, provided by Parto Roshd Novin Company (Iran Growlight). The LED treatments included monochromatic blue light (peak at 460 nm), monochromatic red light (peak at 660 nm), a blue/red combination with a 1:3 ratio, and full-spectrum white light (400–700 nm). A control group received no LED treatment (Fig. 1). The light intensity was maintained at 200 µmol m⁻² s⁻¹ with an 11-hour photoperiod. LEDs were installed directly above the plants, and their distance was adjusted with a PAR meter to ensure uniform light intensity across all plants.

### Stress treatments

Four stress treatments were applied to the plants: control, salinity (80 mM NaCl), alkalinity (40 mM NaHCO₃), and a combined salinity/alkalinity (S/A) treatment. Twenty days after planting, each pot received 100 mL of a solution containing NaCl (for salinity), NaHCO₃ (for alkalinity), or both (for S/A) to ensure consistent stress application. The concentrations of 40 mM NaHCO₃ and 80 mM NaCl were chosen based on preliminary dose-response experiments, which identified these levels as inducing observable stress responses in the plants. This approach allowed for an examination of how different light spectra might alleviate these stress effects. Stress treatments continued for the remaining 60 days of the experiment (total duration: 80 days), leading up to data collection.

### Vegetative growth characteristics

At the end of the experiment, all plants were harvested for biomass analysis. Branches, roots, and crowns were separated, and dry weight was determined by oven-drying the samples at 70 °C for 72 h, followed by weighing. Leaf area was measured by randomly selecting three mature leaves from each treatment and assessing their area using a 202 m-CI leaf area meter.

### Early yield and fruit biochemical characteristics

Once the plants began producing fruit, harvests were conducted, and the fruits were weighed to determine the early yield at the end of the experiment. Strawberries were considered ripe when the dark red color covered more than 75% of the fruit surface, serving as the harvesting indicator. All fruits were collected at this stage. To measure total anthocyanin content, 5 g of fruit was combined with 10 ml of acidic methanol (methanol and hydrochloric acid in a 99:1 volume ratio) in a 50 ml falcon tube and homogenized using an ULTRA-TURRAX model IKT18 basic device. The mixture was then centrifuged for 10 min at 4 °C and 4500 rpm. Next, 1 ml of the extract was mixed with 4 ml each of HCl-KCl buffer (pH 1) and acetate buffer (pH 4.5), and the absorbance of the two solutions was measured using a spectrophotometer at 520 nm and 700 nm [19].

$${\rm{A}}\left( {{\rm{absorption}}} \right){\rm{  =  }}\left( {{\rm{A52}}{{\rm{0}}_{{\rm{pH1}}}}{\rm{ - A70}}{{\rm{0}}_{{\rm{pH1}}}}} \right){\rm{ - }}\left( {{\rm{A52}}{{\rm{0}}_{{\rm{pH4}}{\rm{.5}}}}{\rm{ - A70}}{{\rm{0}}_{{\rm{pH4}}{\rm{.5}}}}} \right)$$

$${\rm{Total\, anthocyanin }}\left( {{\rm{mg/l}}} \right){\rm{  =  }}\left( {{\rm{A/30}}{\rm{.200a}}} \right){\rm{ }}\left( {{\rm{103}}} \right){\rm{ }}\left( {{\rm{433}}{\rm{.2b}}} \right){\rm{ }}\left( {{\rm{15c}}} \right)$$

a = molar extinction coefficient of cyanidine triglycoside.

b = molecular weight of cyanidine triglycoside.

c = dilution factor.

The titratable acidity of the fruit was determined by extracting 3 ml of juice, diluting it with 25 ml of distilled water, and adding 4 drops of 1% phenolphthalein reagent. The solution was titrated with 0.2 N sodium hydroxide until a color change was observed. Fruit acidity was expressed as grams of citric acid per 100 ml of fruit juice [20].

Antioxidant activity was measured using the DPPH assay. For this purpose, 5 g of fruit was homogenized with 10 ml of 80% methanol using an ULTRA-TURRAX IKT18 basic device until a uniform mixture was obtained. The homogenate was then centrifuged at 4 °C for 10 min at 4500 rpm. Subsequently, 100 µL of the extract was mixed with 900 µL of DPPH solution in ethanol and vortexed thoroughly, then incubated in the dark for 30 min. A control sample was prepared in the same manner, except methanol was used in place of the extract. Absorbance was measured using a spectrophotometer at 517 nm. A blank reaction without DPPH was included to correct for background absorbance [21]. The percentage of antioxidant activity was calculated using the following formula:

$${\rm{Antioxidant\:activity}} = 1 - {{\left( \matrix{{\rm{Sample\:number}} -  \hfill \cr {\rm{Correction\:coefficient\:number}} \hfill \cr}  \right)} \over {{\rm{Control\:number}}}} \times 100$$

in which.

Correction coefficient number: the read number related to the mixture of extract and ethanol.

Sample number: the read number corresponding to the mixture of extract and DPPH.

Control number: The number read for the mixture of ethanol and DPPH.

The pH difference method was used to calculate the total phenolic compounds of the fruit. The amount of total phenolic compounds was calculated using the gallic acid standard of 1 mM in mg per 100 g of fresh weight [22].

### Leaf osmotic characteristics

#### Proline measurement

To determine proline concentration, the leaves were homogenized in 5 ml of 95% ethanol, and the insoluble fraction of the extract was separated. The resulting solution was centrifuged at 3500 rpm for 10 min. Then, 1 ml of the supernatant was diluted with 10 ml of distilled water, followed by the addition of 5 ml of ninhydrin reagent (prepared by dissolving 1.25 g of ninhydrin in 30 ml of glacial acetic acid and 20 ml of 6 M phosphoric acid). Afterward, 5 ml of glacial acetic acid was added, and the mixture was heated in a water bath for 45 min. Once cooled, 10 ml of benzene was added, and the solution was mixed with a mechanical stirrer to transfer the proline into the benzene phase. The samples were then left to stand for 30 min before measuring the absorbance at 515 nm using a spectrophotometer. Proline concentration was expressed as µmol g⁻¹ fresh weight of the leaf sample [23].

#### Total phenolic compounds

For measuring total phenolic compounds in leaves, the alcoholic extract prepared earlier for proline analysis was used. One milliliter of the extract was combined with 2.5 ml of 10% (w/v) Folin-Ciocalteu reagent. After 5 min, 2 ml of 5% sodium carbonate was added, and the mixture was incubated at 50 °C for 10 min with intermittent stirring. The samples were then cooled, and absorbance was measured at 765 nm using a spectrophotometer. Total phenolic compounds were quantified against a 1 mM gallic acid standard and expressed as mg/100 g [24].

#### Sucrose

To measure sucrose content, 0.2 ml of the alcohol extract (prepared earlier for proline analysis) was mixed with 0.1 ml of 30% KOH and heated at 100 °C for 10 min. After cooling, 3 ml of anthrone solution (prepared by dissolving 150 mg of anthrone in 100 ml of 72% sulfuric acid) was added, and the samples were incubated at 40 °C for 10–15 min. Once cooled, the absorbance was measured at 620 nm. A standard curve was created using sucrose concentrations of 0, 20, 40, 60, 80, and 100 µg/ml to calculate sucrose content, expressed as mg per g of fresh weight [25].

#### Starch

To measure leaf starch content, 1 g of dried leaf sample was ground into a fine powder. Then, 200 mg of the powdered sample was mixed with 1 ml of distilled water until fully moistened. Next, 5 M NaOH was added, and the mixture was stirred for 1 h. Following this, 3.8 ml of 5 M hydrochloric acid was added, and the solution volume was brought up to 10 ml with deionized distilled water. Subsequently, 1 ml of this solution was diluted with 3 ml of distilled water, and 120 µl of the diluted solution was added to a test tube containing 3.68 ml of sodium phosphate buffer, ensuring the pH was neutral. Then, 0.2 ml of iodine reagent was added, and the mixture was vortexed. After 10 min, absorbance was measured at 600 nm using a spectrophotometer. The starch content was calculated in mg/g dry weight based on the absorbance values of standard solutions [26].

#### Soluble carbohydrates

To measure leaf soluble carbohydrates, 0.1 ml of the alcoholic extract prepared for proline analysis was mixed with 3 ml of freshly prepared anthrone solution (150 mg of anthrone dissolved in 100 ml of 72% sulfuric acid). This mixture was then heated in a water bath at 70 °C for 10 min to allow the reaction to proceed. After cooling, the absorbance was measured at 625 nm using a spectrophotometer, and the concentration of soluble sugars was calculated based on standard values [27].

### Enzyme activity

#### Polyphenol oxidase

In this enzyme method, 100 µl of the enzyme extract was combined with 1.4 ml of the assay solution (containing 5 mM H_2_O_2_, 200 mM phosphate buffer adjusted to pH 5, 100 mM citrate, and catechol at a final concentration of 0.05 M). Enzyme activity was measured using a spectrophotometer at a wavelength of 420 nm for 2 min at 24 °C and expressed as units per mg of protein in the homogenate.

From frozen strawberries, 1.0 g of sample was mixed with 2.0 ml of extraction solution containing 0.5% (v/v) Triton X-100, 100 mM phosphate buffer (pH = 7), and 1.5% (w/v) polyvinylpyrrolidone. The resulting homogenate was centrifuged at 12,000 g for 20 min. After centrifugation, the supernatant was collected for enzyme assay. Polyphenol oxidase activity was measured using pyrogallol as a substrate. The reaction mixture contained potassium phosphate buffer (2.5 mL, 50 mM, pH 7.2), pyrogallol (200 µL, 0.02 mol/L) and 100 µL of enzyme extract. Absorbance at 420 nm was recorded every 5 s for a maximum of 3 min. Enzyme activity was determined using pyrogallol extinction coefficient (6.2 mM^− 1^cm) based on the following formula [28].

$${\rm{A  =  \varepsilon bc}}$$

Enzyme activity was expressed as units (U) of enzyme per mg of total protein in 100 µLof extract [29].

#### Guaiacol peroxidase

To assay guaiacol peroxidase activity, a mixture consisting of potassium phosphate buffer (2.77 mL, 50 mM, pH 7), guaiacol (100 µL, 4%), H_2_O_2_ (100 µL, 1%), and 30 µL of enzyme extract was prepared. The absorbance of the solution at a wavelength of 470 nm was recorded kinetically for 3 min. The extinction coefficient (26.6 mM^− 1^cm^− 1^ at 470 nm) was used to calculate the guaiacol peroxidase activity, where one unit of guaiacol peroxidase was the enzyme that oxidizes 1.0 mmol of tetra-guaiacol per minute [30].

### Mineral elements

The dried leaf and root samples were ashed by heating at 550 °C for 5 h. The resulting ash was dissolved in 5 ml of 2 N HCl, followed by the addition of 50 ml of distilled water. Sodium (Na) and potassium (K) concentrations in the leaves were determined using flame photometry (Jenway, model PFP7). Iron (Fe) and copper (Cu) concentrations were measured with an atomic absorption spectrophotometer (GBC-Savant AA, Australia). Magnesium (Mg) concentrations were measured using EDTA titration [31].

For chloride (Cl) measurement, 1 g of dried sample was ground and mixed with 100 ml of 0.01 M nitric acid, then left overnight. The solution’s pH was adjusted to 7.5–8 with sodium carbonate, and a few drops of potassium chromate reagent were added. The solution was titrated with 0.05 N silver nitrate, and Cl concentration was calculated using the following equation [32]:

$${\rm{Cl }}\left( {{\rm{mg/kg\,dry\,weight}}} \right){\rm{  =  N  \times  ml  \times  5}}{\rm{.35\, Equation\, 11 - 3}}$$

Where: N: normality of silver nitrate and ml: ml of titrated acid.

### Experimental design and data analysis

This experiment employed a completely randomized factorial design with two factors. The first factor, light spectrum, included five levels: monochromatic blue, monochromatic red, a 1:3 blue/red combination, white light, and a control (no LED treatment). The second factor, stress, consisted of four levels: control, salinity, alkalinity, and combined salinity/alkalinity (S/A) treatment. Each treatment combination contained two plants per pot, with three replicates.

Data analysis was conducted using SAS software (version 9.4) with a two-way ANOVA to assess the main and interactive effects of light spectrum and stress on the measured parameters. Significant differences (*P* < 0.01) were identified using the LSD Multiple Range Test following confirmation of significant effects via ANOVA. Pearson’s correlation coefficient was used to evaluate relationships between the studied parameters, and correlation diagrams were generated with Origin Pro software (version 2023b). Graphical representations of the data were created in Excel (version 2016), while principal components analysis (PCA) was performed using XLSTAT software (version 2015.5.01.22537). To better visualize parameter changes under different treatments, a heatmap was generated. Data were first normalized due to varying parameter values, and the heatmap was then created with Origin Pro software.

## Results

### Vegetative growth characteristics

**Fig. 2:**
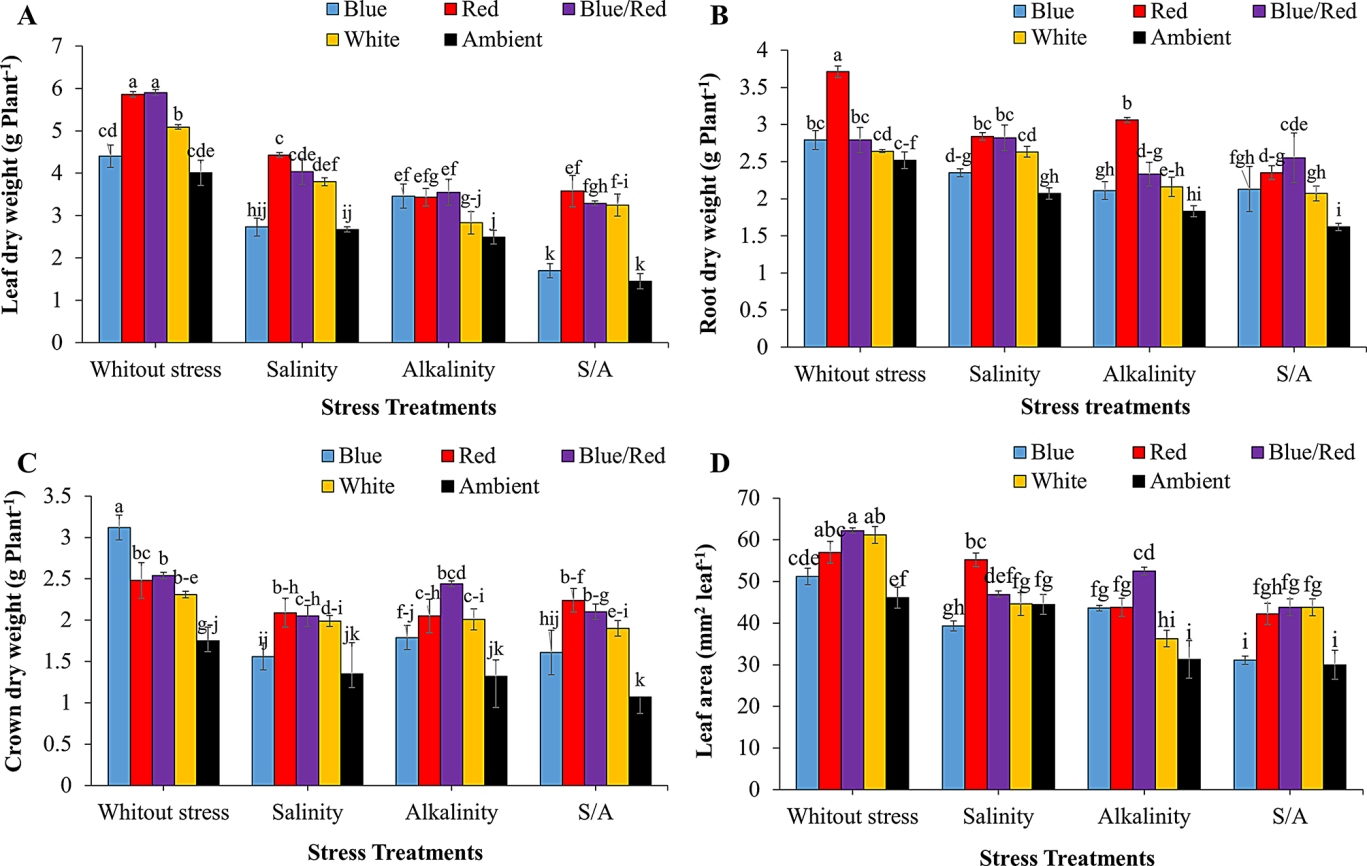
Changes in vegetative growth characteristics of strawberries cv. Sabrina under the effect of five light spectrum levels and four stress levels. **A**: leaf dry weight; **B**: root dry weight; **C**: crown dry weight; **D**: leaf area. Means followed by the same letter for a parameter, are not significantly different according to LSD (*p* ≤ 0.05). Vertical bars indicate the standard errors of three replicates

The dry weights of leaves, roots, and crowns, as well as leaf area, were significantly affected by the interaction between supplementary light and stress treatments (*P* ≤ 0.05) (Table 1). Mean comparisons revealed that stress conditions substantially reduced vegetative characteristics across all supplementary light treatments compared to non-stress conditions. Under salinity and combined salinity/alkalinity stress, red, blue/red, and white light spectra resulted in the highest leaf dry weights. For plants under alkalinity stress, blue, red, and blue/red light treatments significantly increased leaf dry weight, with no significant differences observed among these treatments (Fig. 2A).

Red light also led to a significant increase in root dry weight in both non-stress and alkalinity-stress conditions (Fig. 2B). In non-stress conditions, blue light notably affected crown dry weight. Under salinity and combined salinity/alkalinity stress, red, blue/red, and white light significantly enhanced crown dry weight. Under alkalinity stress, the blue/red light spectrum had the most pronounced effect on crown dry weight (Fig. 2C).

For leaf area, the red and blue/red light spectra significantly increased leaf size under salinity and alkalinity stress conditions, respectively. Under combined salinity/alkalinity stress, the red, blue, and blue/red light spectra all led to a significant increase in leaf area, with no notable differences among these treatments (Fig. 2D).

**Table 1:** ANOVA results of the effect of supplementary light and stress on vegetative growth characteristics and enzyme activity of strawberry plants cv. Sabrina

Source of variations	DF	Mean squares			
		Leaf dry weight	Root dry weight	Crown dry weight	Leaf area
Light (L)	4	6.21^**^	1.58^**^	1.56^**^	377^**^
Stress (S)	3	16^**^	1.58^**^	1.42^**^	854^**^
L × S	12	0.489^**^	0.136^**^	0.213^*^	69.4^**^
Error	40	0.136	0.06	0.084	14.7
CV%		10.2	9.93	14.6	8.47

Significance according to ANOVA, ns, *, **, no significant and significant *P* ≤ 0.05, 0.01, respectively. SAS software version 9.4 was used for data analysis

### Early yield and fruit biochemical characteristics

**Fig. 3:**
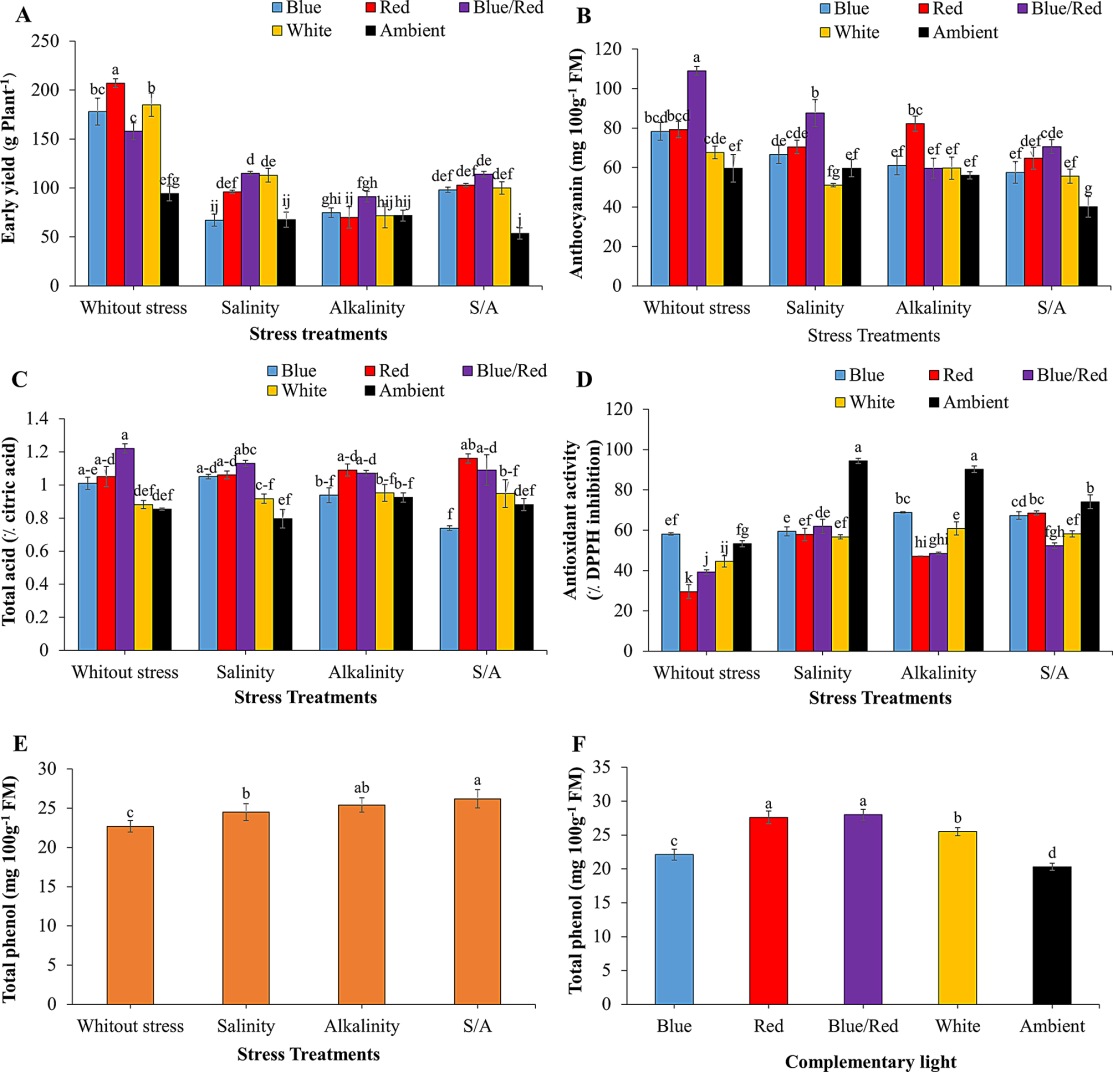
Changes in early yield and biochemicals of strawberries fruit cv. Sabrina under the effect of five light spectrum levels and four stress levels. **A**: early yield; **B**: anthocyanin; **C**: total acid; **D**: antioxidant activity; **E** and **F**: total phenolic compounds. Means followed by the same letter for a parameter, are not significantly different according to LSD (*p* ≤ 0.05). Vertical bars indicate the standard errors of three replicates

The interaction between supplementary light and stress treatments significantly influenced yield, anthocyanin content, total acid, fruit antioxidant activity, and the independent effect of treatments on total phenolic compounds (*P* ≤ 0.05) (Table 2). Mean comparisons showed that salinity, alkalinity, and combined stress conditions significantly reduced strawberry yield compared to non-stress conditions. The highest yield was recorded under red light treatment without stress. Under salinity stress, the blue/red, red, and white light treatments significantly increased yield. In contrast, no significant differences in yield were observed among supplementary light treatments under alkalinity and combined salinity/alkalinity stress (Fig. 3A).

The highest fruit anthocyanin content was observed in non-stress conditions with the blue/red light treatment. In salinity and alkalinity stress conditions, blue/red and red light treatments yielded the highest anthocyanin levels, respectively. Under combined salinity/alkalinity stress, no significant differences were noted among the supplementary light treatments, with the lowest anthocyanin levels found in the treatment without supplementary light (Fig. 3B).

The highest total acid content was recorded in non-stress conditions with blue/red light, while the lowest was under alkalinity stress with blue light (Fig. 3C). Stress conditions increased fruit antioxidant activity, with the highest levels observed in treatments without supplemental light. Supplementary light significantly decreased antioxidant activity compared to no-light treatments under stress conditions (Fig. 3D).

Stress conditions also significantly increased total phenolic compounds in the fruit compared to non-stress treatments (Fig. 3E). Moreover, supplementary light spectra significantly enhanced total phenolic content in the fruit compared to ambient light alone (Fig. 3D).

**Table 2:** ANOVA results of the effect of supplementary light and stress on early yield and biochemical parameters of strawberry fruit cv. Sabrina

Source of variations	DF	Mean squares				
		Early yield	Anthocyanin	Total acidity	Antioxidant activity	Total phenolic compounds
Light (L)	4	5017^**^	1551^**^	0.086^**^	1604^**^	138^**^
Stress (S)	3	23,480^**^	1189^**^	0.004^ns^	1448^**^	34.1^**^
L × S	12	1373^**^	271^**^	0.046^*^	234^**^	7.42^ns^
Error	40	160	86.9	0.021	13.1	4.43
CV%		11.8	13.9	14.8	6.08	8.5

Significance according to ANOVA, ns, *, **, no significant and significant *P* ≤ 0.05, 0.01, respectively. SAS software version 9.4 was used for data analysis

### Leaf osmotic characteristics

**Fig. 4:**
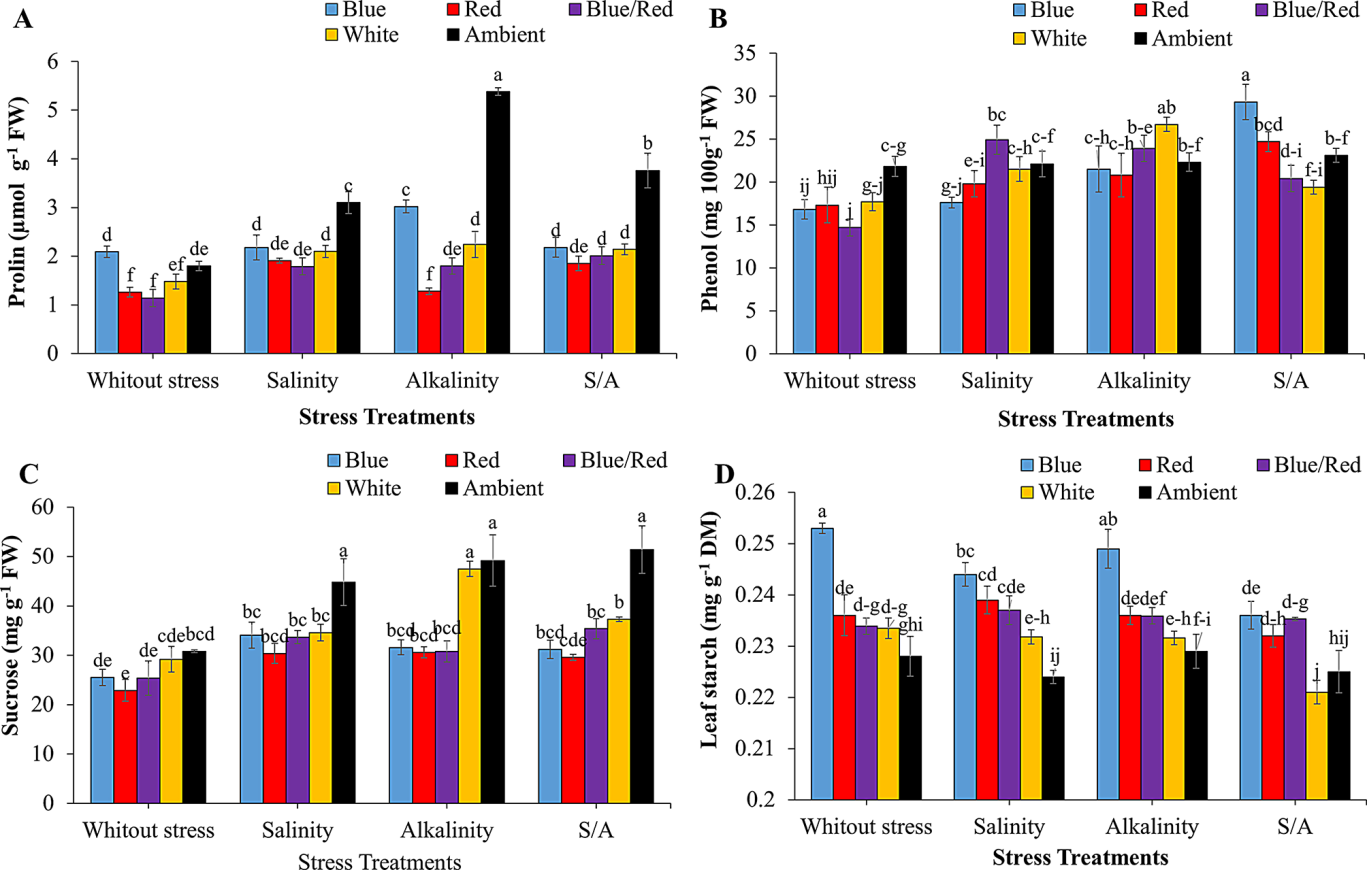
Changes in leaf osmotic regulators of strawberries cv. Sabrina under the effect of five light spectrum levels and four stress levels. **A**: proline; **B**: total phenolic compounds; **C**: sucrose; **D**: leaf starch. Means followed by the same letter for a parameter, are not significantly different according to LSD (*p* ≤ 0.05). Vertical bars indicate the standard errors of three replicates

Proline, total phenolic compounds, sucrose, and starch content in strawberry leaves were significantly affected by the interaction between supplementary light and stress treatments (Table 3). The independent effect of treatments on carbohydrate content was also significant (*P* ≤ 0.01). Stress conditions increased proline, total phenolic compounds, and sucrose levels in the leaves. The highest proline concentration was observed under alkalinity stress with the ambient light treatment (Fig. 4A). Under salinity and alkalinity stress, blue/red and blue light treatments, respectively, significantly increased total phenolic content in leaves compared to other light spectra (Fig. 4B).

Supplementary light treatments significantly reduced leaf sucrose levels under salinity and combined salinity/alkalinity stress compared to ambient light. Specifically, blue, red, and blue/red light treatments markedly decreased leaf sucrose under salinity stress (Fig. 4C). The highest starch content in the crown was recorded under non-stress conditions with blue light, which was not significantly different from blue light treatment under alkalinity stress (Fig. 4D).

**Fig. 5:**
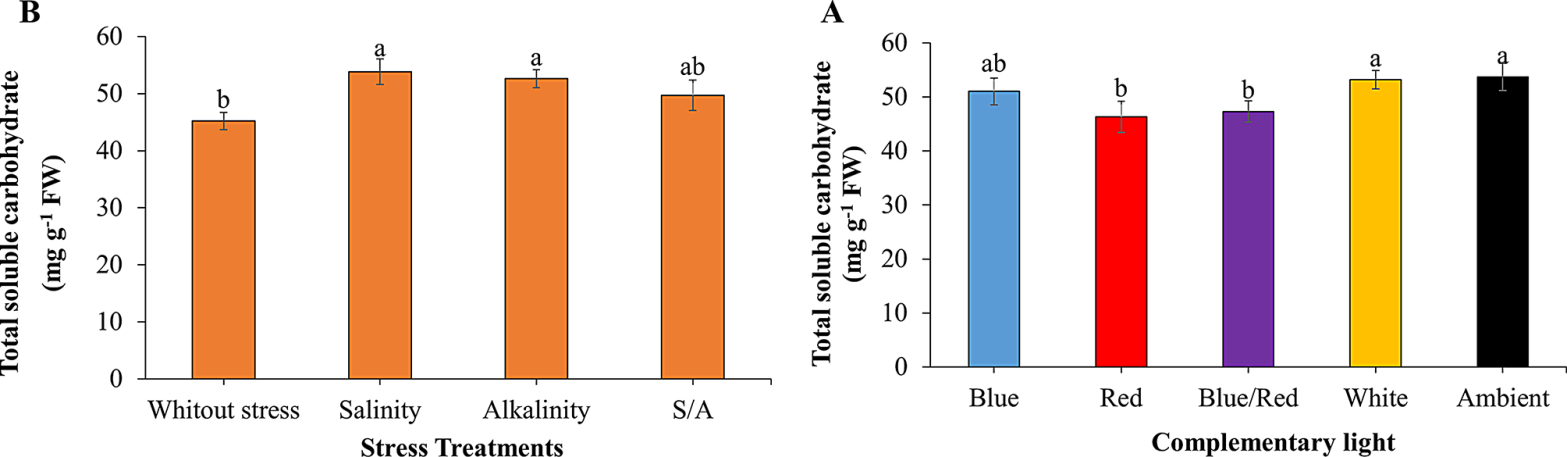
Independent effect of light (**A**) and stress (**B**) on leaf total soluble carbohydrates of strawberries cv. Sabrina. Means followed by the same letter for a parameter, are not significantly different according to LSD (*p* ≤ 0.05). Vertical bars indicate the standard errors of three replicates

Both salinity and alkalinity stress significantly increased leaf soluble carbohydrates (Fig. 5A). However, blue/red and red light treatments significantly reduced leaf soluble carbohydrate content compared to no-light treatment, with no notable difference between the blue light spectrum and other light treatments (Fig. 5B).

**Table 3:** ANOVA results of the effect of supplementary light and stress on leaf osmotic parameters and enzyme activity of strawberry plants cv. Sabrina

Source of variations	DF	Mean squares						
		proline	total phenolic compounds	sucrose	starch	TSS	PPO	POX
Light (L)	4	14.2^**^	4.65^ns^	412^**^	0.0006^**^	138^*^	0.0001^**^	0.093^**^
Stress (S)	3	7.09^**^	101^**^	394^**^	0.0001^**^	221^*^	0.0007^**^	0.118^**^
L × S	12	1.71^**^	33.2^**^	67^**^	0.00003^*^	78^ns^	0.00009^**^	0.018^**^
Error	40	0.065	6.94	20	0.00001	0.049	0.00008	0.001
CV%		10	12.3	13	1.79	7.78	12.8	13.9

Significance according to ANOVA, ns, *, **, no significant and significant *P* ≤ 0.05, 0.01, respectively. SAS software version 9.4 was used for data analysis

TSS: Total soluble carbohydrates of leaf; PPO: Polyphenol oxidase; POX: Peroxidase

### Enzyme activity

**Fig. 6:**
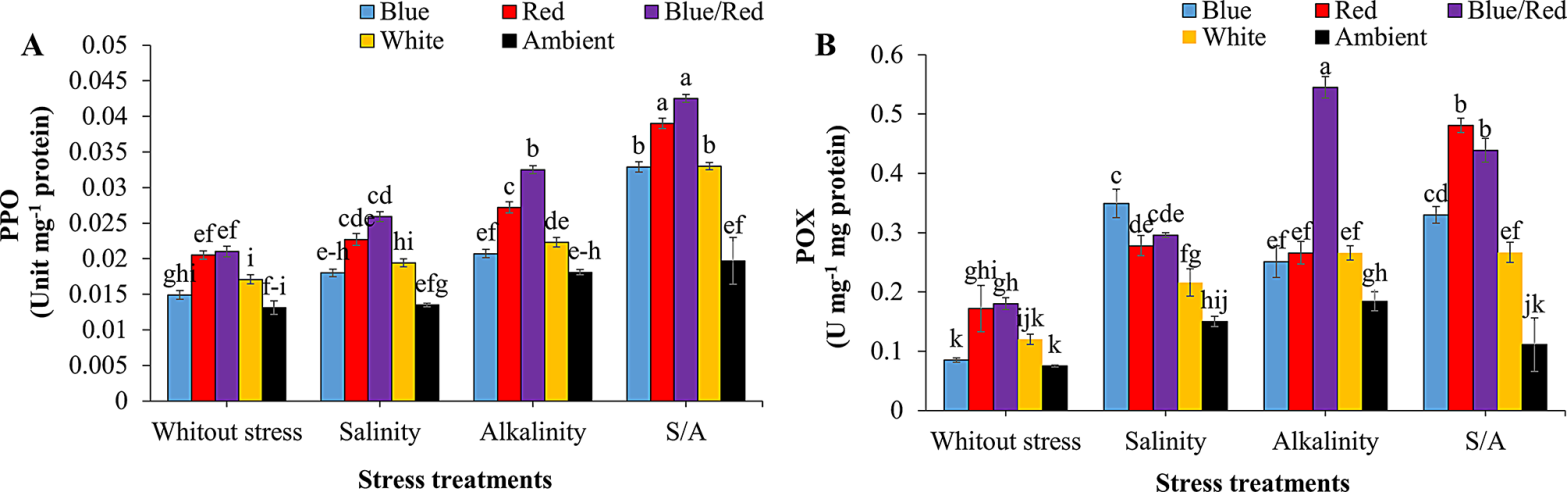
Changes in enzyme activity of leaves of strawberries cv. Sabrina under the effect of five light spectrum levels and four stress levels. **A**: PPO; **B**: POX. Means followed by the same letter for a parameter, are not significantly different according to LSD (*p* ≤ 0.05). Vertical bars indicate the standard errors of three replicates

Polyphenol oxidase (PPO) and peroxidase (POX) enzyme activities were significantly influenced by the interaction between supplementary light and stress treatments (*P* ≤ 0.01) (Table 3). Stress conditions increased the activity of both enzymes. The highest PPO activity was observed under combined salinity/alkalinity stress across all supplementary light treatments (Fig. 6A). For POX activity, the highest levels were found under alkalinity stress with blue/red light treatment. Additionally, under combined salinity/alkalinity stress, red and blue/red light treatments significantly enhanced POX activity compared to other light spectra treatments (Fig. 6B).

### Mineral analysis

**Fig. 7:**
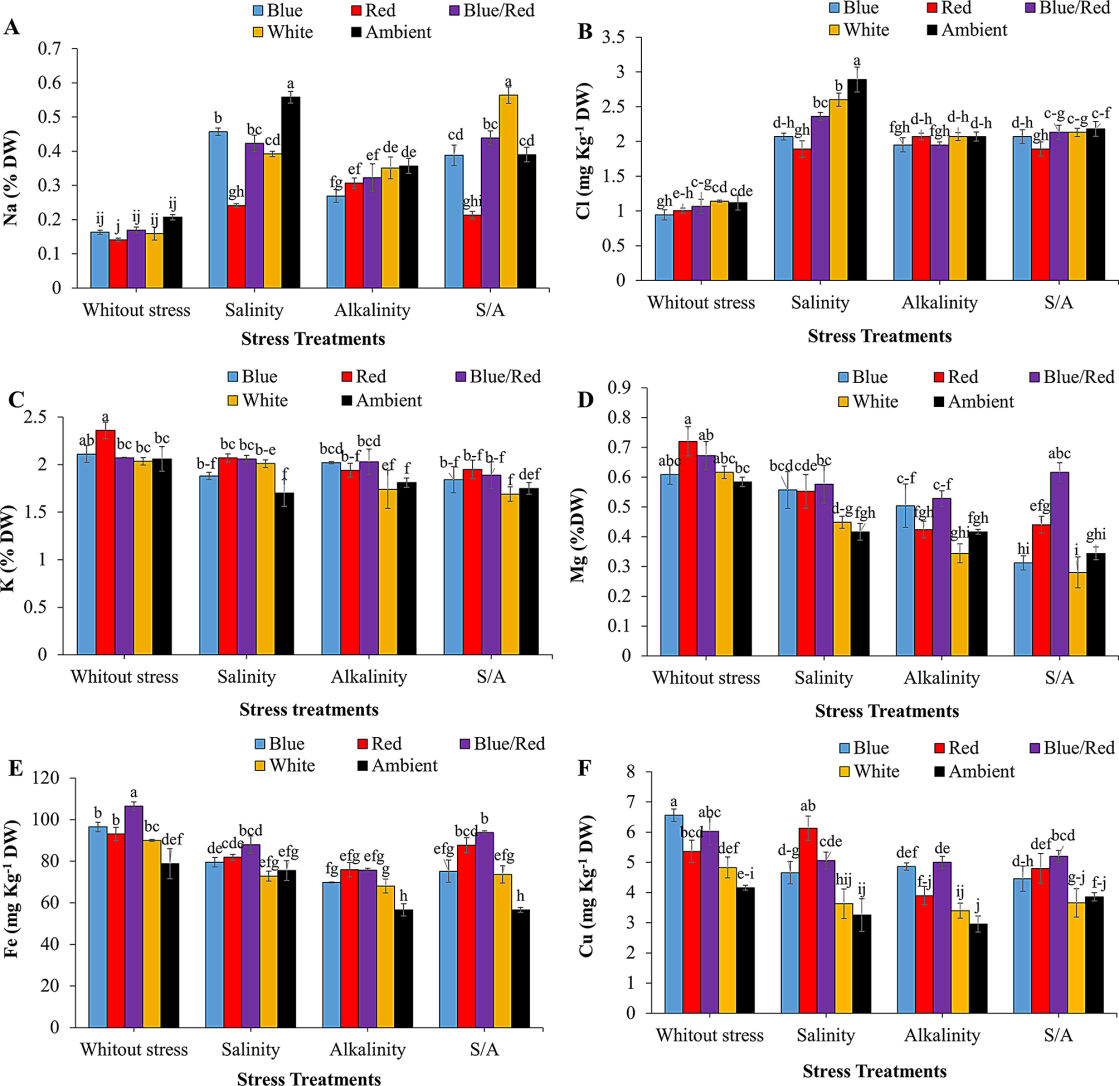
Changes in the mineral elements of leaves of strawberries fruit cv. Sabrina under the effect of five light spectrum levels and four stress levels. **A**: Na; **B**: Cl; **C**: K; **D**: Mg; **E**: Fe; **F**: Cu. Means followed by the same letter for a parameter, are not significantly different according to LSD (*p* ≤ 0.05). Vertical bars indicate the standard errors of three replicates

The concentrations of Na, Cl, K, Mg, Fe, and Cu in strawberry leaves were significantly affected by the interaction between supplementary light and stress treatments (*P* ≤ 0.05) (Table 4). Mean comparisons revealed that stress conditions increased Na and Cl levels in the leaves. Under salinity stress, ambient light treatment resulted in the highest Na accumulation, with no significant difference from the white light spectrum under combined salinity/alkalinity stress. The lowest Na concentration was observed under non-stress conditions, with no significant differences across light spectrum treatments (Fig. 7A). Salinity stress significantly elevated Cl levels in leaves treated with blue/red, white, and ambient light compared to other treatments, while no significant differences were observed among light treatments under alkalinity and combined salinity/alkalinity stress (Fig. 7B).

Stress conditions led to reductions in K, Mg, Fe, and Cu levels in the aerial parts of the plants. The highest shoot K concentration was observed under non-stress conditions with red light, while the lowest concentration occurred under ambient light treatment (Fig. 7C). The blue/red light spectrum significantly increased shoot Mg concentration compared to other light treatments under combined salinity/alkalinity stress (Fig. 7D). The highest Fe content in leaves was recorded under non-stress conditions with blue/red light. In alkalinity stress conditions, complementary light spectra significantly increased Fe content compared to ambient light, with red and blue/red light treatments resulting in the greatest Fe accumulation under combined salinity/alkalinity stress (Fig. 7E).

Under salinity stress, red and blue/red light treatments significantly increased Cu levels in leaves compared to white and ambient light treatments. The lowest Cu content in leaves was observed under ambient light in alkalinity stress conditions, with no significant differences from white and ambient light treatments under the other stress conditions (Fig. 7F).

**Table 4:** ANOVA results of the effect of supplementary light and stress on the concentration of nutrient elements of leaf of strawberry plants cv. Sabrina

Source of variations	DF	Mean squares					
		Na	Cl	K	Mg	Fe	Cu
Light (L)	4	0.018^**^	0.167^**^	0.022^ns^	0.047^**^	549^**^	7.8^**^
Stress (S)	3	0.19^**^	0.318^**^	0.243^**^	0.166^**^	1456^**^	5^**^
L × S	12	0.024^**^	0.165^**^	0.06^**^	0.016^**^	221^**^	0.82^**^
Error	40	0.001	0.023	0.027	0.004	32.6	0.368
CV%		10.6	7.21	8.52	14	7.15	4.82

Significance according to ANOVA, ns, *, **, no significant and significant *P* ≤ 0.05, 0.01, respectively. SAS software version 9.4 was used for data analysis

### Principal component analysis

After standardizing the data to achieve a zero mean and unit variance, principal component analysis (PCA) was conducted to examine changes in 22 parameters across five light spectrum treatments and four stress levels. The magnitude of each vector in the PCA plot reflects the influence of the corresponding parameter, while its direction is determined by PCA1 and PCA2 values. The PCA analysis visualized changes in the 22 parameters under the five light spectrum treatments in the following conditions: (A) no stress, (B) salinity stress, (C) alkalinity stress, and (D) combined salinity/alkalinity stress.

**Fig. 8:**
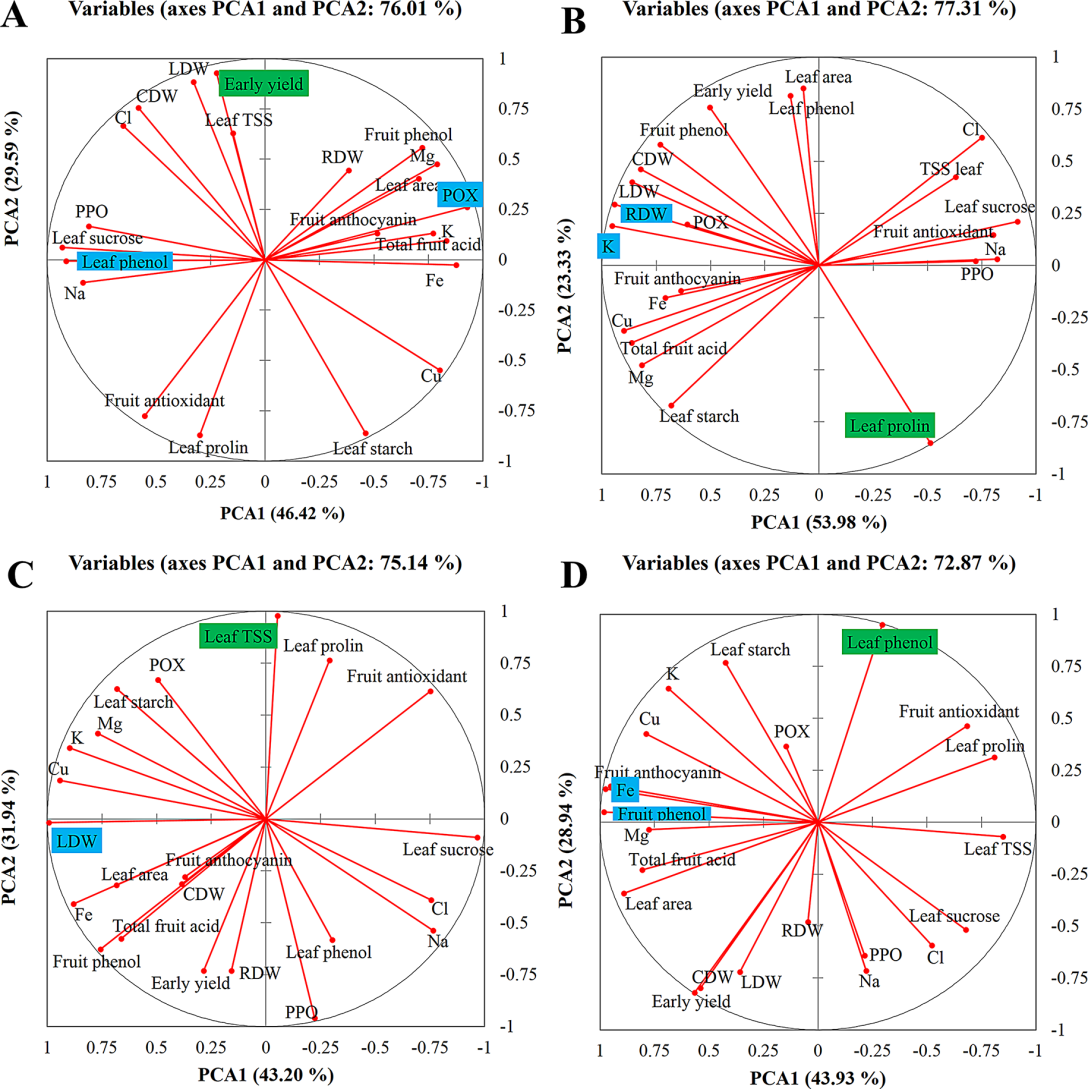
Vector graphs show the relative “contribution” of each input variable to the formation of the principal components. Principle component biplot based on variations of five levels of light spectra under (**A**) control (without stress); (**B**) salinity stress; (**C**) alkalinity stress; (**D**) salinity/alkalinity stress. Principal component analysis and biplots were performed using XLSTAT software version 2015

In the control (no stress) condition, PCA1 and PCA2 explained 76.01% of the total variance among the light spectrum treatments (Fig. 8A). For salinity, alkalinity, and combined salinity/alkalinity stress treatments, the percentages of explained variance were 77.31%, 75.14%, and 72.87%, respectively (Fig. 8B and C, and 8D).

Under control conditions with all five light spectrum treatments, leaf total phenolic compounds and polyphenol oxidase (POX) were the primary contributors to PCA1, while early yield made the largest contribution to PCA2. In the salinity stress treatment, potassium (K) content and root dry weight were the main contributors to PCA1, with leaf total phenolic compounds most influencing PCA2. For alkalinity stress, leaf dry weight was the primary contributor to PCA1, while leaf total soluble solids (TSS) were most influential for PCA2. In the combined salinity/alkalinity stress treatment, leaf iron (Fe) and fruit total phenolic compounds had the strongest impact on variability across the light spectrum treatments.

The blue and green highlights represent the factor or factors that contributed the most in PCA1 and PCA2, respectively.

### Heatmap diagram and correlation analysis

A heatmap was created using color gradients to illustrate changes in measured parameters across five light treatments and four stress levels. Values ranged from red (highest values) to green (lowest values), highlighting key parameters with significant variations. Notable changes included fruit antioxidant and leaf sucrose levels under ambient light, especially in salinity and alkalinity stress conditions. Additionally, polyphenol oxidase (PPO) activity was a critical parameter in non-stress conditions with ambient light. Under the blue/red spectrum without stress, fruit anthocyanin content was prominent, while potassium (K) levels were notably affected in the red light treatment without stress. In combined salinity/alkalinity (S/A) stress, peroxidase (POX) activity, fruit acid content, and total phenolic compounds were significant in red light treatments. Total sugar, starch, and POX activity were key indicators under blue light with alkalinity stress. Chloride (Cl) content was a major factor under salinity stress in both white and ambient light, whereas sodium (Na) had the greatest impact under S/A stress in white light treatments.

**Fig. 9:**
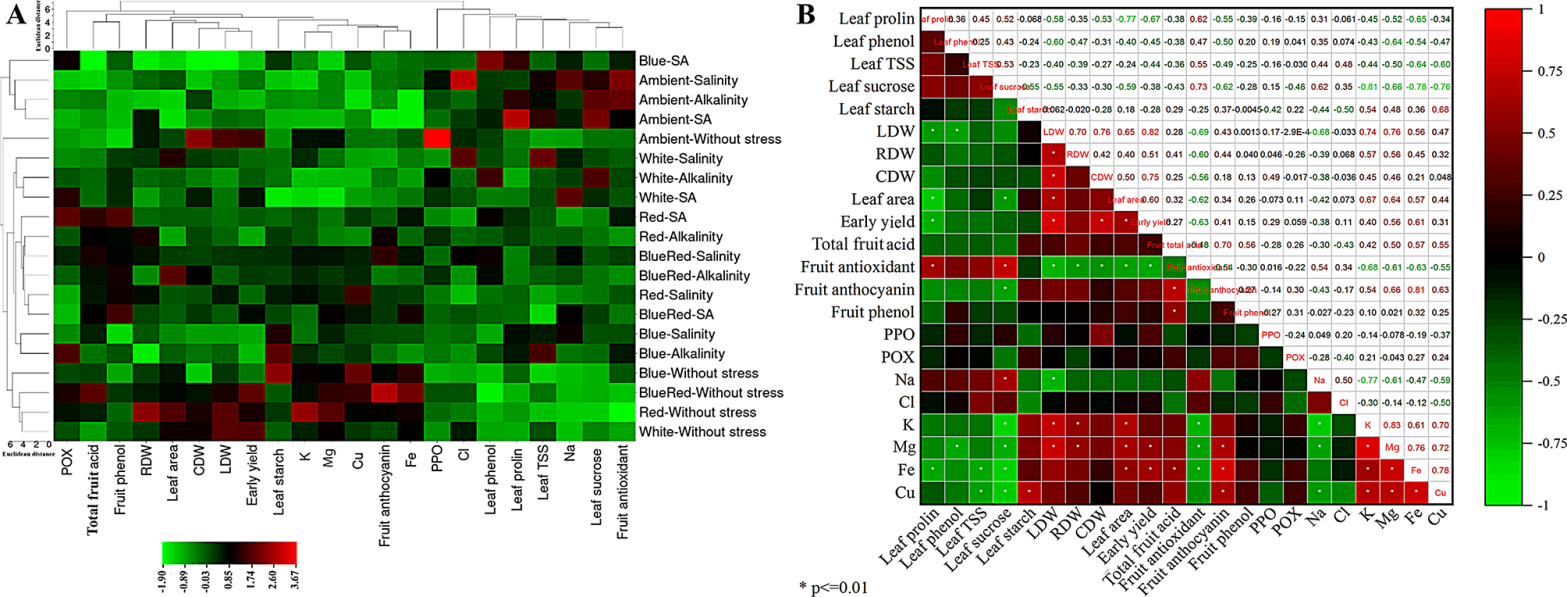
Heatmap diagram of treatments clustering in rows and measured traits in columns (**A**) and correlation plot between the measured traits (**B**)

In general, under non-stress conditions, vegetative growth, yield parameters, and elements such as K, Fe, and Cu were most impactful. In contrast, enzyme and antioxidant activities were critical under stress conditions. The heatmap categorized treatments into two main groups: (1) ambient light under stress and blue/red spectrum under S/A stress, and (2) all other treatments. Within the second group, ambient light without stress formed a separate subgroup, while other treatments clustered together. Cluster analysis further grouped measured traits into two categories: the first included Na and Cl content, PPO activity, antioxidant activity, and leaf osmotic regulators; the second comprised vegetative growth, yield parameters, K, Mg, Fe, Cu, and fruit quality traits (Fig. 9A).

The correlation diagram (Fig. 9B) showed positive correlations between K, Mg, Fe, and Cu with vegetative growth parameters, while these elements negatively correlated with proline, total phenolic compounds, total soluble carbohydrates, and leaf sucrose content. Conversely, Na and Cl were positively correlated with proline, total phenolic compounds, total soluble carbohydrates, leaf sucrose, and fruit antioxidant activity. Collar starch exhibited positive correlations with K, Mg, Fe, Cu, and vegetative growth and yield characteristics.

## Discussion

Reductions in shoot and root dry weight are key indicators of plant stress sensitivity [33]. A common response to salt stress includes limiting leaf area and restricting growth [34]. Both salinity and alkalinity stress cause leaf chlorosis, degrade chlorophyll, and decrease photosynthesis, ultimately reducing leaf water content [35, 36]. These factors collectively hinder growth and biomass production. Plants detect changes in light quality via photoreceptors that regulate growth and development through specific signaling pathways. Light quality and intensity profoundly influence nutritional, physiological, and morphological traits [37]. In this study, increased dry weight under blue/red light correlated with expanded leaf surface area and higher leaf counts [38]. Under stress conditions, red and blue/red light treatments maximized leaf area, underscoring their significance for growth under salinity and alkalinity stress. Strawberry yield reduction under salt stress is linked to decreased fruit count and size [39], and light quality plays a role in enhancing fruit size [40]. Previous studies demonstrate that combining blue and red light increases strawberry fruit production by promoting glucose transfer from leaves to fruit under LED lighting [9, 10]. Alkalinity stress can reduce sugar content due to high pH disrupting root function and causing ion imbalances [41].

Plant secondary metabolites, such as total phenolic compounds, are involved in light signaling and defense against abiotic stress [42]. Blue and red light are particularly effective in enhancing anthocyanin biosynthesis compared to darkness [43]. The association between blue light receptors and anthocyanin accumulation is well-established; for instance, blue light activates phototropins, which are linked to increased anthocyanin levels [44, 45]. Blue light also elevates total phenolic compounds in crops like tomatoes and grapes [46, 47], while combined blue and red light enhances anthocyanin in greenhouse crops [48]. Starch synthesis occurs during photosynthesis and serves as a storage carbohydrate [49]. Stress conditions in this study led to reduced starch content, possibly due to disruptions in ADP-glucose pyrophosphorylase activity, resulting in starch conversion to soluble sugars [50]. This breakdown contributes to osmotic adjustment, helping plants maintain osmotic balance under stress [51]. Blue/red light supplementation in the study improved crown starch content, likely by enhancing photosystem II efficiency [52].

To counter environmental stress, plants accumulate osmotic compounds like proline, total phenolic compounds, and soluble carbohydrates to maintain cellular functions [53]. The heatmap (Fig. 9A) emphasizes the roles of osmotic regulators, enzymes, and antioxidants in stress response. Proline accumulation is a key physiological adaptation to stress, aiding in osmotic balance and mitigating free radical damage [54]. Stress conditions, such as salinity and alkalinity, trigger proline biosynthesis through the activity of glutamine kinase [55]. Total phenolic compounds also play essential roles in metabolism and stress defense, especially under oxidative stress, helping to detoxify reactive oxygen species (ROS) [56, 57]. The heatmap (Fig. 8) highlights the importance of total phenolic compounds and soluble carbohydrates in stress adaptation. Accumulation of total phenolic compounds is a non-enzymatic defense mechanism aiding plants in resisting oxidative damage [58], with similar findings reported in strawberries under salinity stress [59]. In response to salinity and alkalinity, plants break down larger molecules like starch into simple sugars to maintain osmotic balance and prevent dehydration [60]. Stress conditions alter carbohydrate metabolism during photosynthesis, increasing soluble carbohydrates [61], and similar adaptive responses are observed in crops like sorghum [62].

Photoreceptors, such as cryptochromes (CRY1–CRY3), phytochromes (PHYA, PHYE), and phototropins (PHOT1, PHOT2), regulate plant responses to light, affecting processes like anthocyanin biosynthesis and chlorophyll production [63, 64]. Antioxidant enzymes, including catalase (CAT), superoxide dismutase (SOD), and ascorbate peroxidase (APX), help mitigate oxidative stress [65]. Red and blue light enhance antioxidant capacity in various crops, such as lettuce, rice, tomatoes, basil, and buckwheat sprouts [66–70]. This study aligns with these findings, indicating that LED lighting promotes the synthesis of total phenolic compounds and enhances antioxidant capacity.

Salinity and alkalinity stress negatively impact nutrient absorption and transport [71]. High sodium concentrations cause osmotic and metabolic issues, reducing biomass production [72]. Alkalinity can render minerals insoluble, particularly iron, zinc, and copper, impairing chlorophyll synthesis and causing leaf chlorosis [73]. Potassium levels decrease under bicarbonate stress due to sodium antagonism [74], and magnesium, essential for chlorophyll, is reduced under stress [75, 76]. In this study, salinity and alkalinity stress decreased magnesium in shoots, likely due to sodium competition [77], and salinity stress inhibited iron transport, as previously noted in citrus, likely from disrupted root proton pump activity [78, 79]. Blue, red, and combined light spectra improved mineral content in strawberry plants. Blue light, through phototropin activation, promotes ion transport by opening plasma membrane ion channels [80–82], supporting findings that blue light enhances mineral accumulation in various plants [83, 84]. Red light also aids nutrient uptake via phytochrome pathways that stimulate root growth [85]. This study confirmed that combining red and blue light optimizes nutrient absorption under both stressed and non-stressed conditions.

## Conclusion

The impact of various supplementary light sources on plant growth, nutrient content, and stress tolerance holds practical importance for horticulture. Our study demonstrated that strawberry plants exposed to blue, red, and especially blue/red complementary light showed improved tolerance to stress conditions. This underscores the need for research across different plant species to evaluate their resilience under diverse supplementary light treatments. The analysis of physiological, morphological, and mineral elements in strawberry plants indicated that they utilize distinct coping mechanisms for abiotic stress, depending on the light quality received. Specifically, blue and red-light spectra were found to enhance antioxidant activity and nutrient uptake, which contributed to improved vegetative and reproductive growth. These light spectra also increased plant resistance to stress, allowing for greater tolerance under adverse conditions. This research has practical applications for optimizing plant growth and development in challenging environments, as selecting the appropriate light quality can alleviate the negative impacts of stress. By strategically enhancing light conditions, horticulturists can boost plant resilience and overall productivity under abiotic stress conditions.

## Data Availability

The authors declare that data supporting the findings of this study are available in the article. If raw data files are required, they are available from the corresponding author upon reasonable request.
